# Causal associations of BAFF-R on IgD+ CD24- B cell immune cell trait with hepatocellular carcinoma and the mediating role of phenylacetylglutamate levels: a Mendelian randomization study

**DOI:** 10.7150/jca.96059

**Published:** 2024-06-24

**Authors:** Xuan Zhu, Zongchao Qiu, Maochun Yang, Jiali Yang, Lingxi Kong, Limin Li, Yingting Huang, Li Xie

**Affiliations:** 1 Department of Medical Laboratory, The Second Affiliated Hospital of Guangxi Medical University, Nanning 530007, Guanxi, China.; 2 Fujian Yuanshizhuling Community Health Service center, Jiangnan district, Nanning 530031, Guanxi, China.; 3 Department of Occupational and Environmental Health, Beihai Center for Diseases Prevention and Control (Beihai Institute of Health Supervisio), Beihai, 536000, Guanxi, China.

**Keywords:** Mendelian randomization, immune cell, BAFF-R on IgD+ CD24- B cell, hepatocellular carcinoma, phenylacetylglutamate

## Abstract

We conducted a bi-directional two-sample Mendelian randomization (MR) analysis to investigate the causal associations between immune cell traits and hepatocellular carcinoma (HCC) and identified the mediating factor of metabolites. The exposure factors were immune cell traits, the mediators were metabolites, and the outcome variable was HCC. Inverse-variance weighted method (IVW) was the main method. Weighted median, MR-Egger regression, weighted mode, simple mode, and MR pleiotropy residual sum and outlier (MRPRESSO) methods were used as complementary methods. The results were tested by using the Bayesian weighted Mendelian randomization (BWMR) approach in our MR study. Subsequently, the potential mediating effect was investigated by conducting a two-step mediation analysis. We identified 26 traits with suggestive correlations between immune cell traits and HCC, with 4 immune cell traits among them having causal correlations with HCC. There were no causal correlations between HCC and immune cell traits in the reverse MR analysis. In the mediation analysis, we found a positive causal association between B cell-activating factor receptors (BAFF-R) on IgD+ CD24- B cell and HCC [IVW: odd ratio (OR), 0.845; 95% CI, 0.759-0.942; p = 0.002]. Phenylacetylglutamate (PAG) levels mediated 7.353% of the causal pathway from BAFF-R on IgD+ CD24- B cell and HCC. In conclusion, BAFF-R on IgD+ CD24- B cell lowers risk of HCC, with PAG levels playing a mediating role.

## Introduction

Hepatocellular carcinoma (HCC) accounts for more than 90% of primary liver cancers, with a male-to-female ratio of 2.8:1 1. It ranks sixth among the common cancers but is the fourth leading cause of cancer-related death globally due to its poor prognosis and high recurrence rate 2. It is predicted that by 2025, new cases of HCC will exceed 1 million per year 2, 3. The subtle onset of HCC has led to over 50% of patients being diagnosed at an advanced stage, necessitating systemic therapy as the primary treatment option 4. Significant progress has been made in first-line drugs from tyrosine kinase inhibitors (such as sorafenib) to immune checkpoint inhibitors, and then the integration of immune checkpoint inhibitors into combination immunotherapy resulted in significant synergies, representing the future trajectory of HCC therapy 5. However, the post-treatment survival outcomes for some patients remain suboptimal 6. It has been suggested that HCC has a complex and highly heterogeneous immune microenvironment.

The tumor microenvironment (TME) of HCC is composed of stromal cells, endothelial cells, hepatic stellate cells, and several immune cells 7. HCC commonly developed from chronic inflammation triggered by viral infection or fatty liver, where immune cells undergo functional reprogramming, leading to impaired immune responses and forming a cancer-prone microenvironment 7, 8. Complex metabolic-immune cross-talk is a characteristic feature of HCC 9. Altered metabolic pathways, including metabolites that act as oncogenes or tumor suppressors, are involved in the pathogenesis of HCC 10. Metabolic reprogramming in the TME of HCC can lead to metabolic rearrangement of immune cells, resulting in their own dysfunction and ultimately resulting in immune evasion by tumor cells 9. The relationship between metabolites and the function of immune cells in the TME is inseparable. The FDA has approved the application of small-molecule metabolites for treating specific tumors, making targeted therapy for metabolites a promising approach for treating HCC 11. While numerous clinical trials and Mendelian randomization (MR) analyses have been conducted to investigate the correlation between immune cells and HCC, no study has examined metabolites as mediators of this relationship. However, confirming the causalities between immune cells and HCC, and whether metabolites play a mediating role, is challenging due to potential confounders and measurement errors. Additionally, considering ethical concerns, it is not feasible to measure causal relationships in experimental settings.

MR analysis is similar to randomized controlled trials, using single nucleotide polymorphisms (SNPs) as instrumental variables (IVs) to infer causal relationships between exposure and outcomes 12. Alleles are randomly separated during meiosis without interference from external factors, and genetic variation occurs before the disease. MR can reduce the bias caused by confounding factors and avoid the interference of reverse causality 13, 14. This method is also widely used in various clinical causal inferences 15, 16. However, to date, there is no MR analysis focusing on the association of immune cells, metabolites, and HCC. This study is the first to conduct a mediation analysis to investigate the potential mediating effect of metabolites on the causal associations between immune cells and HCC. Our study offers a new perspective for finding potential targets and developing new and effective treatment methods for HCC.

## Materials and Methods

### Study design

**Figure 1:**
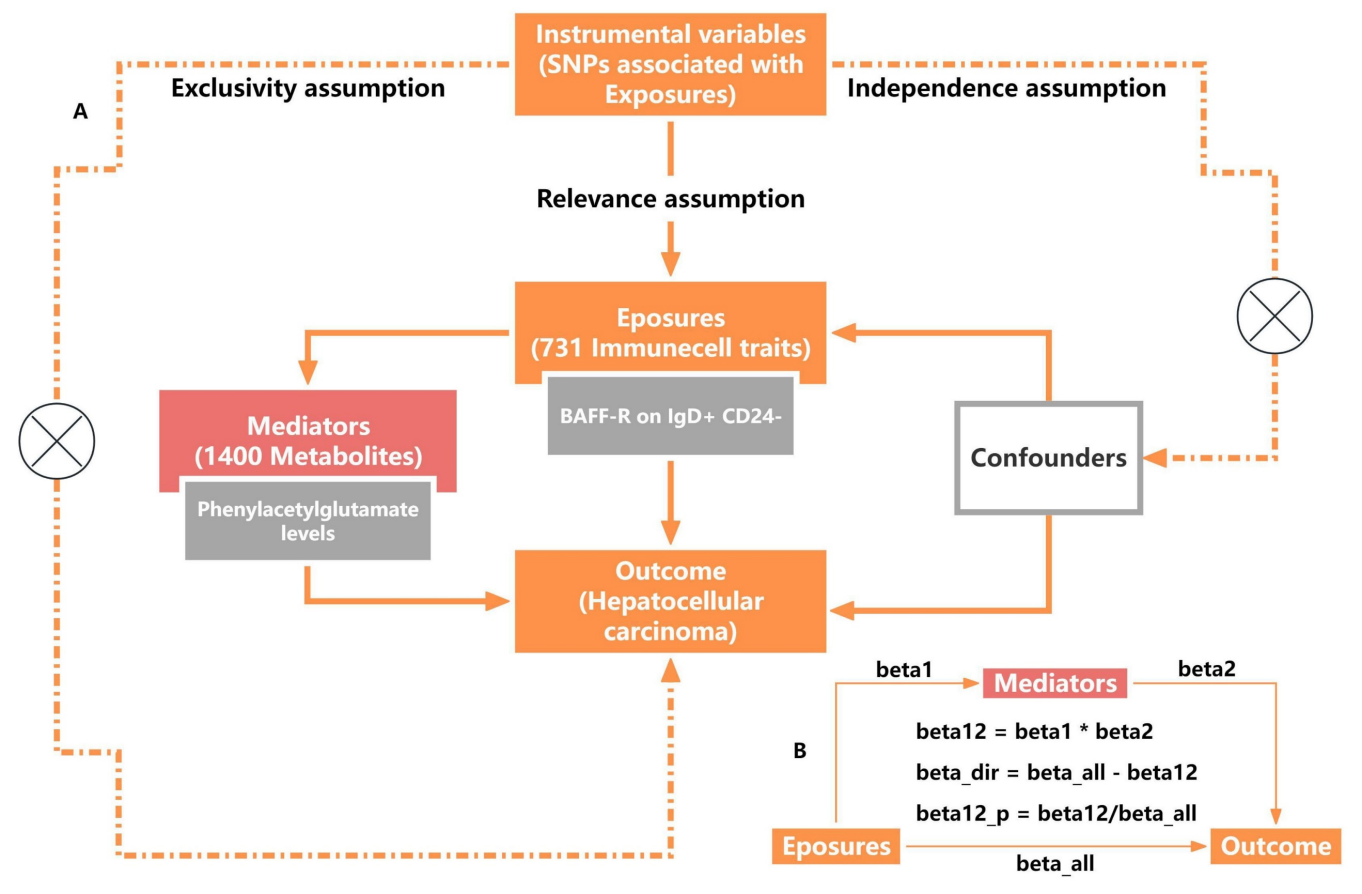
**Study disign overview. (A)** Three critical assumptions of MR analysis. **(B)** Mediation analysis. All effects were beta_all, midiation effect was beta12, dirction effect was beta_dir, the proportion of mediating effect in the total effect was beta12_p.

A two-sample Mendelian randomization (TSMR) method was used (Figure 1A) to assess the causality of 731 immune cell traits on the risk of HCC and a two-step mediation analysis was used to investigate whether this relationship could be mediated by 1400 metabolites. MR studies must follow three fundamental assumptions: **I)** Relevance assumption: the IVs should be closely associated with exposure. **II)** Independence assumption: IVs should be independent of potential confounders. **III)** Exclusivity assumption: IVs impact outcomes only via exposure. The analysis was performed in four steps. We first investigated the overall causal association between immune cell traits and HCC using bidirectional TSMR analyses, then identified the significant immune cell traits causally associated with HCC. Next, we utilized the most significant immunophenotypes as exposures to identify significant mediating factors among 1400 metabolites. Then, the most significant metabolites related to immune cell traits were used as mediating factors to analyze the causality of HCC. Finally, we performed mediation analysis to calculate the mediating effect of phenylacetylglutamate (PAG) levels in the causal pathway from B cell-activating factor receptors (BAFF-R) on IgD+ CD24- B cell to HCC.

### Data sources

The accession numbers of immune cells data were from GCST0001391 to GCST0002121, obtained from the GWAS Catalog provided by Valeria Orrù's group 17. In this study, they profiled 731 immune cell traits and genotyped 22 million SNPs from 3,757 European individuals of unique family-based cohort, based on the high-density arrays or imputed through sequence-based reference panel. The whole 731 cell traits included 389 median fluorescence intensities (MFI) of surface antigens, 118 absolute cell (AC) counts, and 32 morphological parameters (MP). Data was acquired through 7 panels: TBNK (T cells, B cells, natural killer cells) panel, regulatory T cell (Treg) panel, maturation stages of T-cell panel, dendritic cell (DC) panel, myeloid cell panel, monocyte cell panel, and B cell panel. The MP feature contains cDC and TBNK panels. Data on metabolites were obtained from the GWAS Catalog with accession numbers from GCST90199621 to GCST90201020, comprising 1,091 metabolites and 309 metabolite ratios from 8,299 individuals of the Canadian Longitudinal Study on Aging (CLSA) cohort (published PMID: 36635386) 18. HCC data was obtained from the FinnGen database, including 456,348 individuals (453 cases with European ancestry and 287137 controls with European ancestry). The number of SNPs was 20167509. Diagnostic and inclusion criteria were based on the original literature.

The bias caused by confounding factors was minimized since no participants had sample overlap between the exposure and outcome traits. As this study was conducted based on publicly available data, no ethics approval was needed.

### Instrument selection

The genetic variation was selected as an instrumental variable using the following steps to choose the best SNPs correlated with exposure factors. First, all P values were set to 5×10^-8^ to identify independent SNPs. The statistical significance threshold was adjusted to P < 1×10^-5^ to identify more SNPs of both immune cell traits and metabolites as exposures in the forward MR analysis and prevent inaccurate results due to insufficient SNPs. The significance levels was also adjusted to P < 5×10^-5^ to identify more SNPs of HCC as exposures in the reverse MR analysis. The threshold was set to r^2^ < 0.001 within a 10000 kb distance to avoid linkage disequilibrium (LD) between the selection of SNPs. The IVs were screened in the Phenoscanner database (http://www.phenoscanner.medschl.cam.ac.uk/) to identify eligible SNPs and exclude confounders related to HCC risk, including cirrhosis, hepatitis B infection, and hepatitis C infection, hepatitis D infection, Non-alcoholic fatty liver diseases (NAFLD), chronic ingestion of aflatoxin-contaminated foodstuffs, alcohol addiction, obesity, smoking, and type II diabetes. The threshold of LD was set to r^2^ < 0.001 within a 10000 kb distance. The F statistic was calculated using the approximation method to assess the statistical strength between the IVs and the exposure. An F value less than 10 suggests a weak correlation within the IVs, which means that a gene variant with low decoding ability to exposure provides limited statistical power for testing the hypothesis. It may lead to inaccurate estimates of causal effects and an elevated likelihood of type I errors. SNPs with F ≤ 10 were automatically excluded. r^2^ is the explained variance of IVs.

### Mendelian randomization and statistical analysis

TSMR study was performed to assess the causal relationship between 731 immune cell traits (exposure) and HCC (outcome) using the “TwoSampleMR” (version 0.5.7) package of R (version 4.3.1). In this study, the inverse variance weighted (IVW) method was the main method with odd ratios (ORs) to describe the increase in risk levels per standard deviation (SD). Considering the possible heterogeneity and pleiotropy of SNPs, MR-Egger, weighted median, and MR-PRESSO methods were used as supplements to verify the causal effect. The weighted median is the best choice in the presence of heterogeneity and the absence of horizontal pleiotropy. It provides an accurate causal estimate when <50% of the instruments are invalid 19. The MR-Egger method provides reliable results when there is potential horizontal pleiotropy, but it is less effective compared with the IVW method 20. The M-PRESSO method was used to detect and, if needed, correct for horizontal pleiotropy and potential outliers 21. Heterogeneous effects among instrumental SNPs in the IVW method were assessed using Cochran's Q test. Finally, leave-one-out analysis was employed in which single variants were successively removed. It assessed whether a single genetic variant had a disproportionate impact on the over-MR estimate. Genetic variation effect estimates were assessed using forest plots. P value was set to 0.05. We defined the IVW method as the primary approach, with p < 0.05 considered as a suggestive feature. And the estimated causal effect of the specific immune cell was considered statistically significant when all three methods, including IVW, weighted median, MR-Egger yield p < 0.05.

### BWMR analysis

The Bayesian weighted Mendelian randomization (BWMR) method was introduced as a complement to MR analysis. This approach considers the uncertainty arising from the polygenic architecture of diseases or traits that are not accounted for in MR analysis 22. It utilizes Bayesian weighted outlier detection methods to address the issue of pleiotropy, which contradicts the IV assumption. Consequently, it effectively resolved the complexities arising from the polygenic nature and pleiotropy of complex immune cell traits. This led to enhanced the reliability and stability of the MR results.

### Mediation analysis

In mediation analysis, the exposure must have a causal association with the outcome, and the total effects of the exposures on HCC were estimated using univariable MR. Furthermore, the exposure must have a causal association with the mediator but not the other way around, with the effect is calculated as beta1. The mediator must have a causal association with the outcome independent of the exposure, with the effect is calculated as beta2. The direct (without mediators) and mediating effects (through mediators) of mediators between the exposure and the outcome were decomposed using TSMR analysis, ensuring that the direct effect and indirect effect of the exposure on the outcome should align in the same direction. The mediating effect was calculated using the following formula: beta12 = beta1*beta2. The proportion of mediating effect in the total effect was calculated using the following formula: beta12_p = beta12/beta_all*100%. The effect of exposure on outcome was considered to be a direct effect and was calculated using the following formula: beta_dir = beta_all - beta12 (Figure 1B).

## Results

### Selection of IVs

All features of SNPs with significant correlations between BAFF-R on IgD+ CD24- B cell, PAG levels, and HCC are shown in Supplementary Tables (see Supplementary Table S3-S6 online). SNPs with LD and ambiguity were eliminated, and MR-PRESSO analysis was used to remove the outlier SNPs. Finally, independent SNPs without proxies were extracted. Whether outcome was HCC or PAG, there were 22 significant SNPs in BAFF-R on IgD+ CD24- B cell immune cell traits which were found as exposure IVs with an F-statistic of 141.123 (see Supplementary Table S3 and S5 online). We considered 59 SNPs in HCC as exposure IVs with an F-statistic of 20.702 (see Supplementary Table S4 online). In total, 23 significant SNPs in PAG levels were considered as exposure IVs with an F-statistic of 24.497 (see Supplementary Table S6 online).

### Associations of immune cell traits with HCC

**Figure 2:**
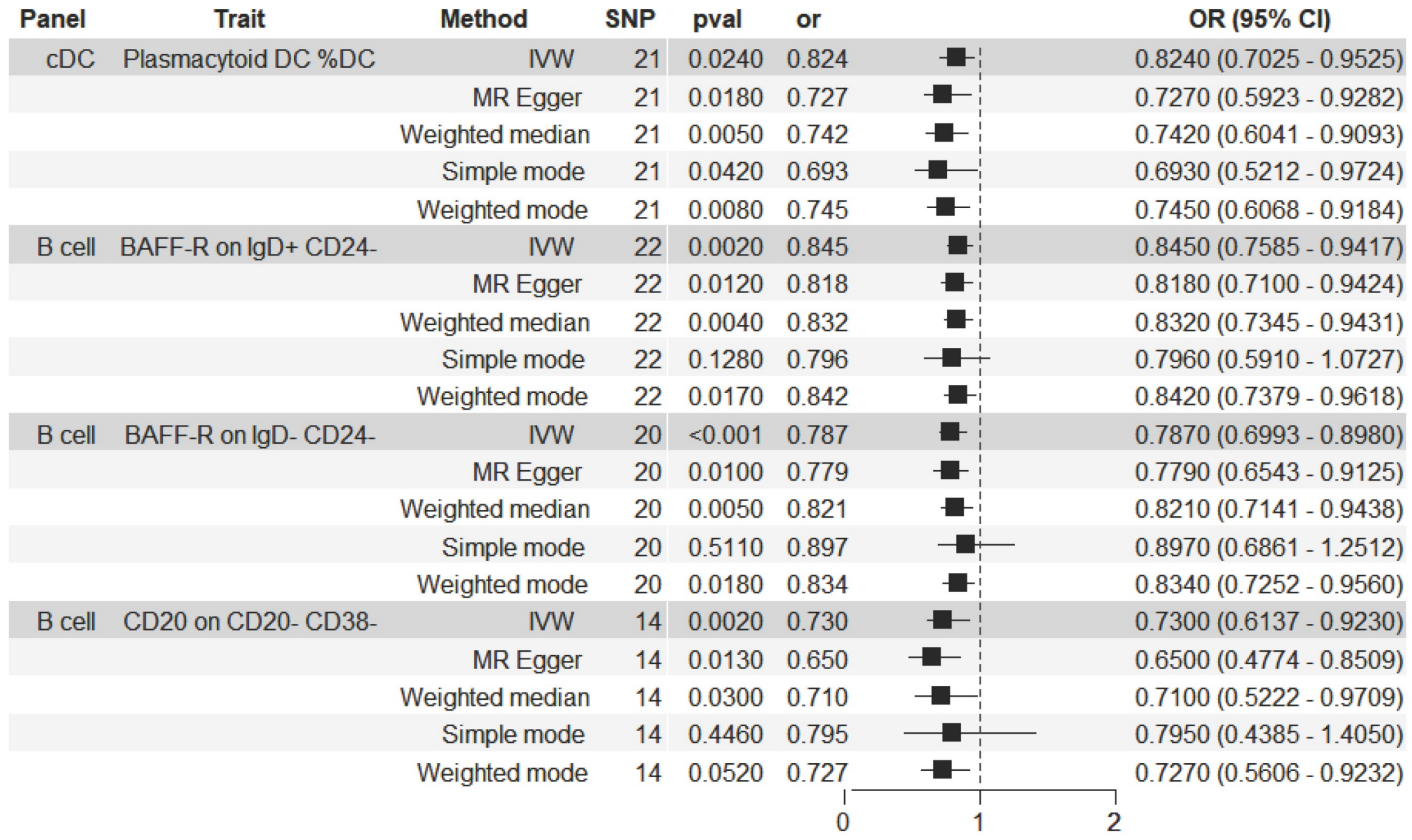
Forest plot illustrating the causal effects between immune cell traits and hepatocellular carcinoma as determined by five MR analyses.

**Figure 3:**
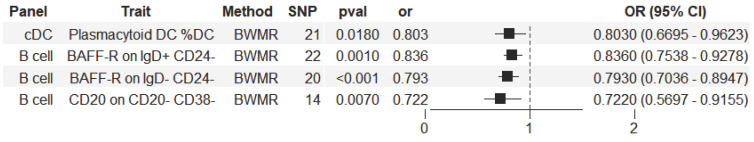
Forest plot illustrating the causal effects between immune cell traits and hepatocellular carcinoma as determined by BWMR analyses.

The significant relationship of immune cell traits on HCC risk were estimated (see Supplementary Table S1 online). According to our definition of suggestive feature and causal effect, there were 26 immune cell traits that exhibited suggestive significant relationship with HCC (P<0.05), of which 14 were in B cell panel, 4 in TBNK panel, 2 in the maturation stages of T cell panel, 3 in Treg panel, 2 in cDC panel, and 1 in myeloid cell panel. Furthermore, the results of MR analysis revealed that 4 increased immune cell traits were identified to have causal associations with a lower risk of HCC (Figure 2). In the cDC panel, one trait stood out: Plasmacytoid DC %DC (IVW: OR, 0.824; 95% CI, 0.696-0.975; P = 0.024; MR Egger: OR, 0.727; 95% CI, 0.572-0.925; P = 0.018; Weighted median: OR, 0.742; 95% CI, 0.603-0.913; P = 0.005; Simple mode: OR, 0.693; 95% CI, 0.497-0.965; P = 0.042; Weighted mode: OR, 0.745; 95% CI, 0.614-0.905; P = 0.008). In the B cell panel, the following were observed: I) BAFF-R on IgD+ CD24- (IVW: OR, 0.845; 95% CI, 0.758-0.942; P = 0.002; MR Egger: OR, 0.818; 95% CI, 0.71-0.942; P = 0.012; Weighted median: OR, 0.832; 95% CI, 0.734-0.944; P = 0.004; Weighted mode: OR, 0.842; 95% CI, 0.74-0.958; P = 0.017), II) BAFF-R on IgD- CD24- (IVW: OR, 0.787; 95% CI, 0.693-0.893; P = 0.0002; MR Egger: OR, 0.779; 95% CI, 0.657-0.923; P = 0.01; Weighted median: OR, 0.821; 95% CI, 0.714-0.943; P = 0.005; Weighted mode: OR, 0.834; 95% CI, 0.727-0.956; P = 0.018), III) CD20 on CD20- CD38- (IVW: OR, 0.73; 95% CI, 0.599-0.891; P = 0.002; MR Egger: OR, 0.65; 95% CI, 0.486-0.868; P = 0.013; Weighted median: OR, 0.71; 95% CI, 0.521-0.968; P = 0.03). Additionally, the BWMR results were shown in Figure 3: Plasmacytoid DC %DC: P = 0.018, BAFF-R on IgD+ CD24-: P = 0.001, BAFF-R on IgD- CD24-: P = 0.0002, CD20 on CD20- CD38-: P = 0.007.

**Figure 4:**
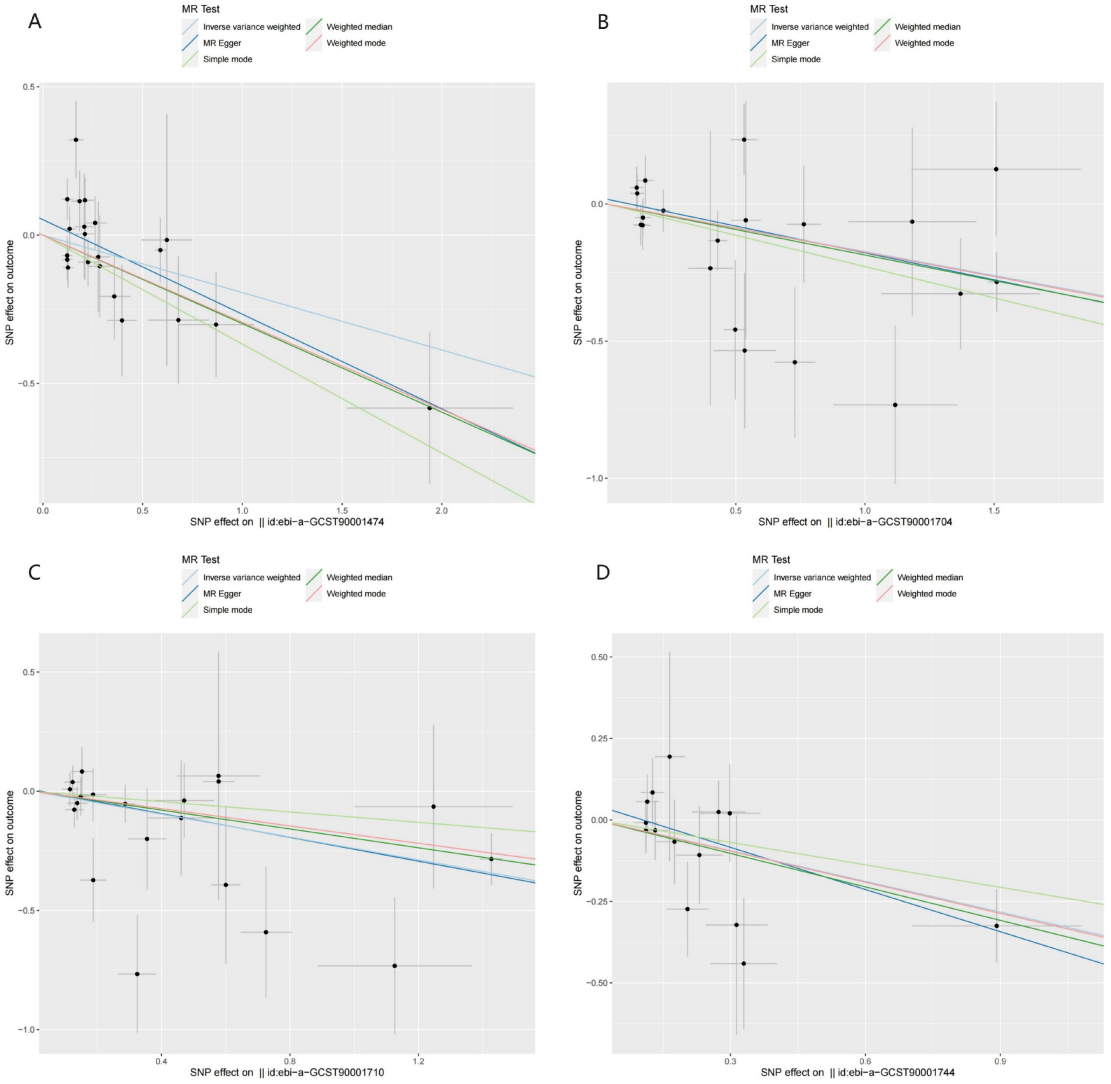
Scatter plots of the MR analyses for the associations of immune cell traits and the risk of hepatocellular carcinoma. A. Plasmacytoid DC %DC; B. BAFF-R on IgD+ CD24-; C. BAFF-R on IgD- CD24-; D. CD20 on CD20- CD38-.

**Figure 5:**
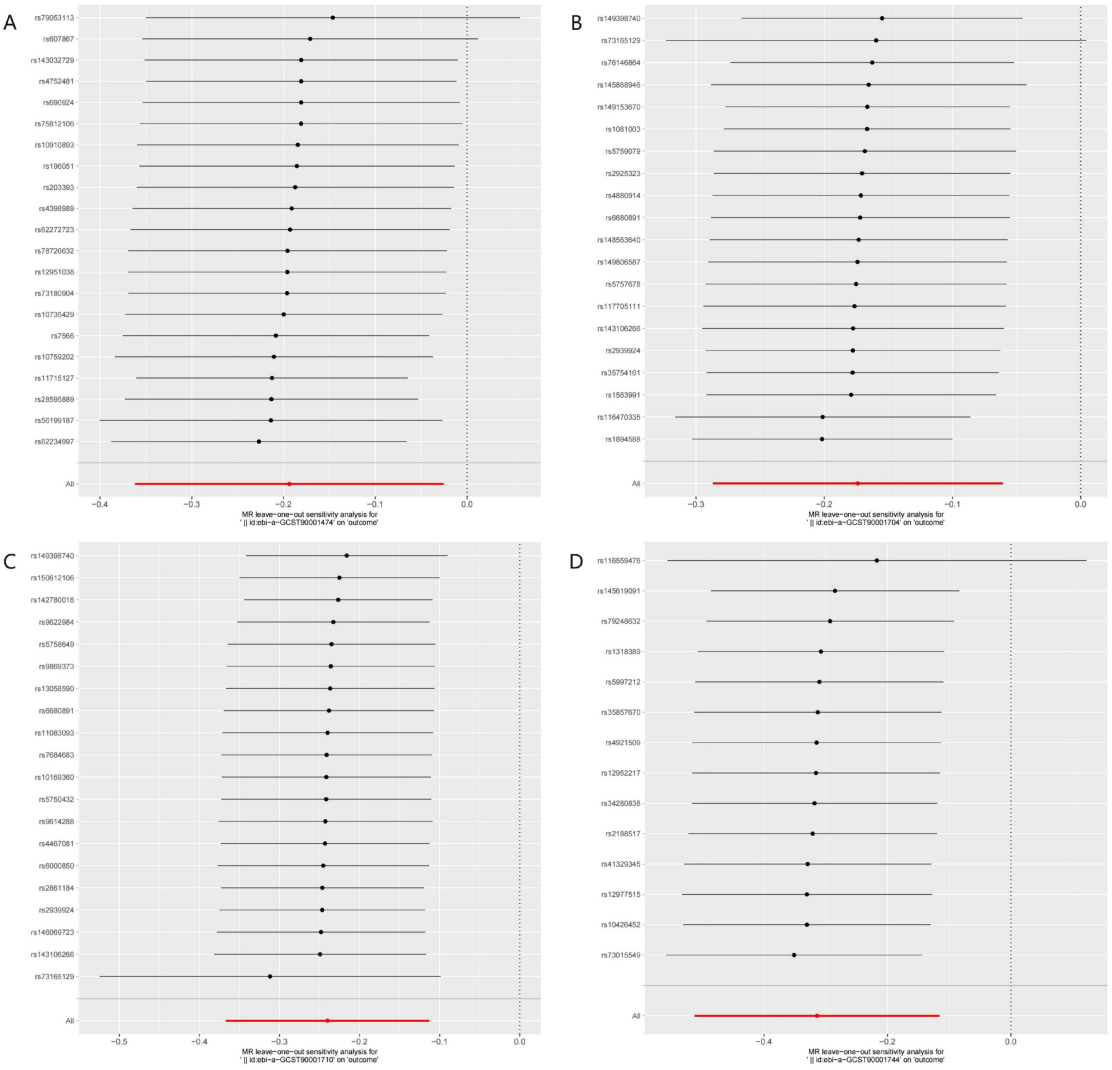
Leave-one-out plots of the MR analyses for the associations of immune cell traits and the risk of hepatocellular carcinoma. A. Plasmacytoid DC %DC; B. BAFF-R on IgD+ CD24-; C. BAFF-R on IgD- CD24-; D. CD20 on CD20- CD38-.

Figure 4 illustrated the causal effect of each IV on HCC. And showed in Figure 5, a leave-one-out sensitivity analysis was conducted. Several sensitivity analyses were conducted to assess the pleiotropy and heterogeneity in causality estimates (see Supplementary Table S1 online). The results of Cochran's Q-test showed no evidence of heterogeneity in the causal associations among these SNPs. The potential pleiotropy was also not detected by the MR-Egger intercept and MR-PRESSO global test (Table 1). All these above analyses supported our MR results. There were no any causal associations in the reverse TSMR analysis between HCC and immune cell traits (see Supplementary Table S2 online).

Firstly, BAFF-R on IgD- CD24- was identified as the most significant immune cell trait. Then, a series of significant metabolites related to BAFF-R on IgD- CD24- were identified. However, there were no positive associations between these significant metabolites and HCC risk. Then, the immune cell trait of BAFF-R on IgD+ CD24- B cell was selected for subsequent mediation analyses.

### Causal effects of BAFF-R on IgD+ CD24- B cell on HCC

**Figure 6:**
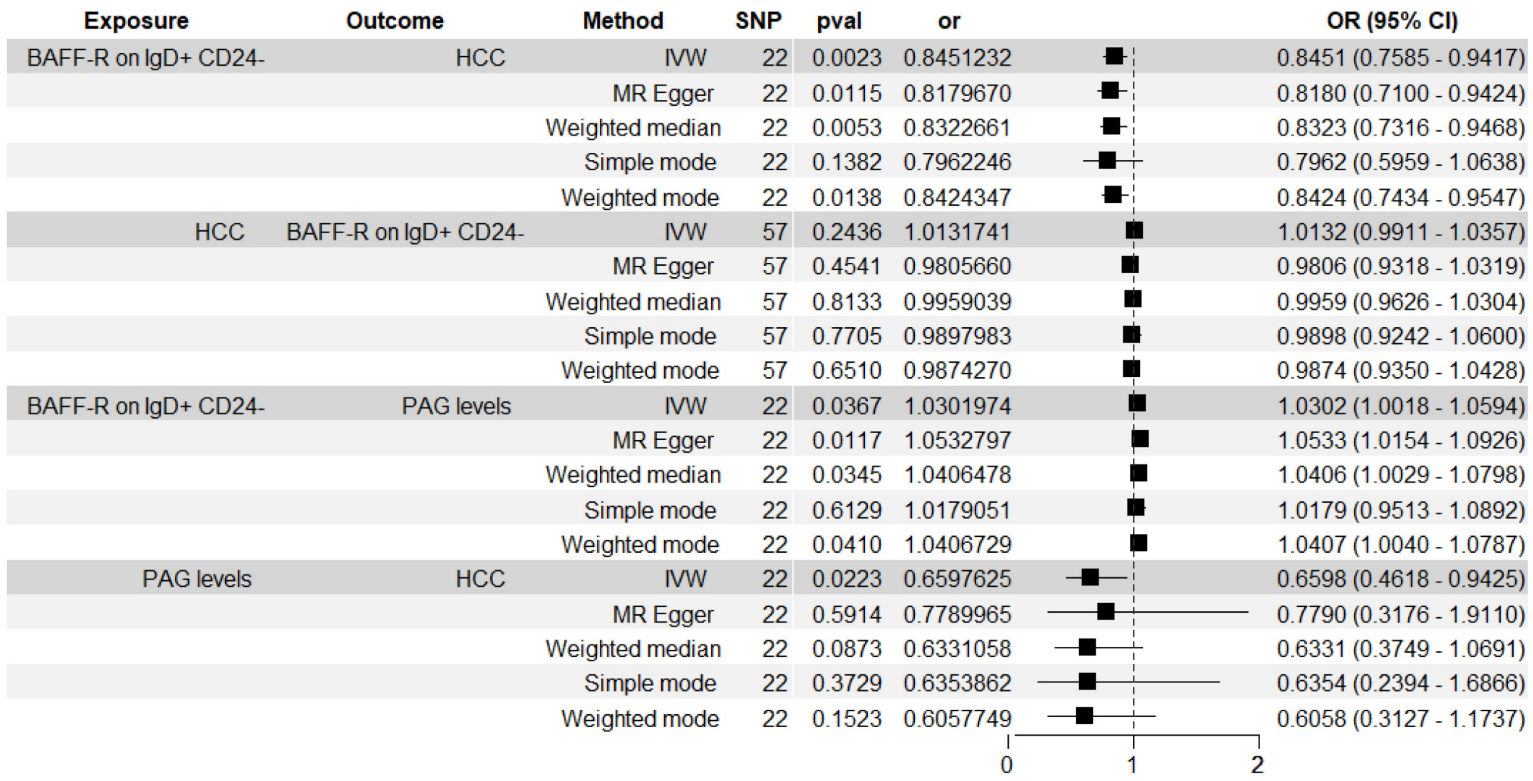
Forest plot illustrating the causal effects between immune cell traits and hepatocellular carcinoma as determined by MR mediation analyses.

Figure 6 showed the associations between BAFF-R on IgD+ CD24- B cell and HCC risk measured by five methods. The BWMR result was show in Figure 3. The significant causal relationship between higher BAFF-R on IgD+ CD24- B cell and lower HCC risk was consistently supported by five MR methods including IVW, BWMR, MR Egger, Weighted median and Weighted mode.

**Figure 7:**
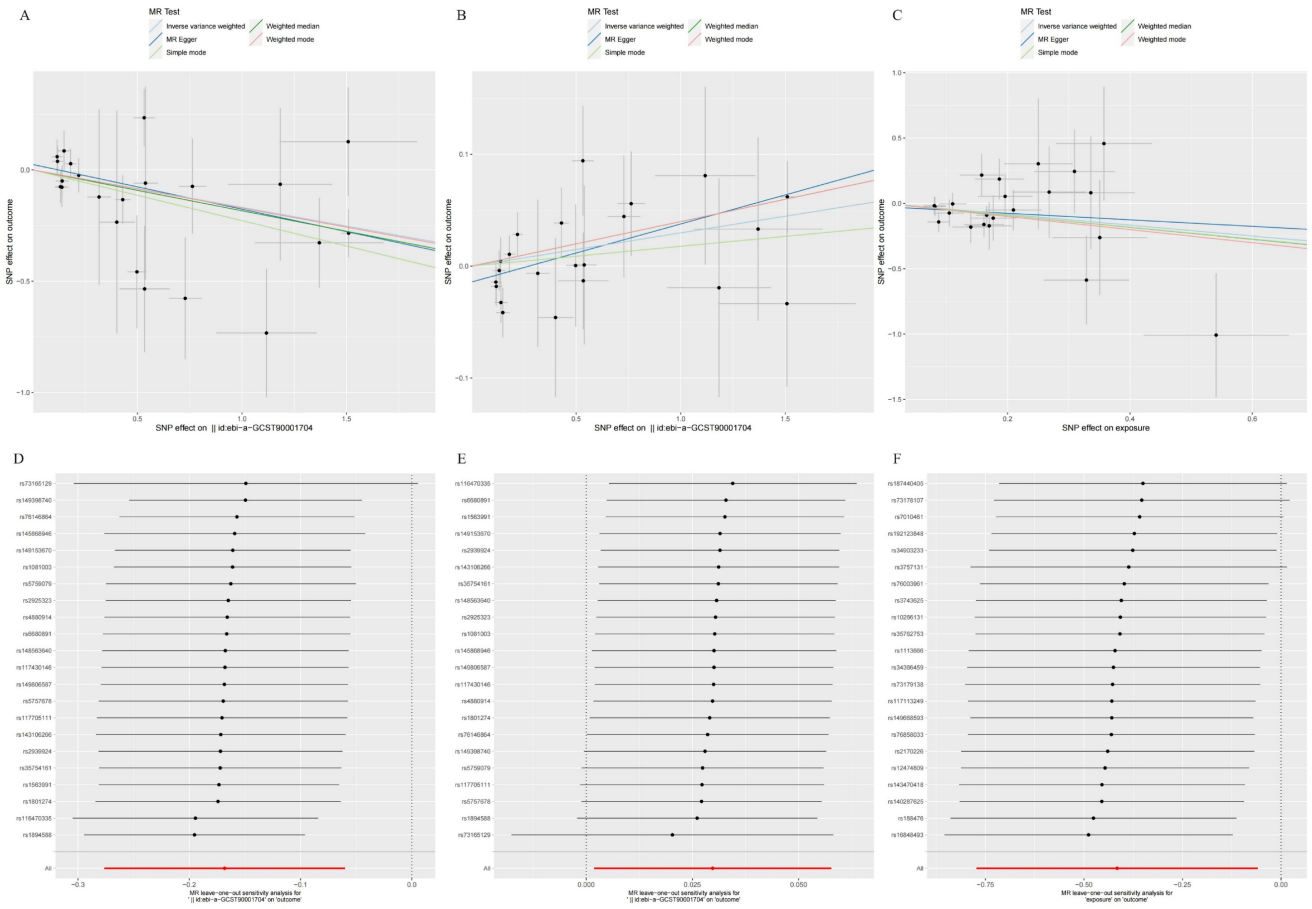
** A.** Scatter plot of MR mediation analysis of BAFF-R on IgD+ CD24- and HCC risk; **B.** Scatter plot of MR mediation analysis of BAFF-R on IgD+ CD24- and PAG levels; **C.** Scatter plot of MR mediation analysis of PAG levels and HCC risk; **D.** Leave-one-out Plot of MR mediation analysis of BAFF-R on IgD+ CD24- and HCC risk; **E.** Leave-one-out plot of MR mediation analysis of BAFF-R on IgD+ CD24- and PAG levels; **F.** Leave-one-out plot of MR mediation analysis of PAG levels and HCC risk. HCC, hepatocellular carcinoma; PAG, phenylacetylglutamate.

Table 1 showed the sensitivity analyses including pleiotropy, heterogeneity and horizontal pleiotropy between BAFF-R on IgD+ CD24- B cell and HCC risk. Scatter plot in Figure 7A visualized the causal effect of BAFF-R on IgD+ CD24- B cell on HCC risk. Leave-one-out analysis indicated that the error line remained relatively stable, suggesting the reliability of our findings (Figure 7D).

### Causal effects of BAFF-R on IgD+ CD24- B cell on PAG levels

Figure 6 showed the associations between BAFF-R on IgD+ CD24- B cell and PAG levels. We found the causal relationship between higher BAFF-R on IgD+ CD24- B cell and higher PAG levels (IVW: OR, 1.03; 95% CI, 1.002-1.059; P = 0.037; MR Egger: OR, 1.053; 95% CI, 1.015-1.093; P = 0.012; Weighted median: OR, 1.041; 95% CI, 1.001-1.082; P = 0.044; Weighted mode: OR, 1.041; 95% CI, 1.003-1.08; P = 0.047).

Table 1 showed the sensitivity analyses including pleiotropy, heterogeneity and horizontal pleiotropy between BAFF-R on IgD+ CD24- B cell and PAG levels. Scatter plot in Figure 7B visualized the causal effect of BAFF-R on IgD+ CD24- B cell on HCC risk. Leave-one-out analysis showed that the correlation of BAFF-R on IgD+ CD24- B cell with PAG levels was not driven by any single SNP (Figure 7E).

### Causal effects of PAG levels on HCC

Figure 6 showed the associations between PAG levels on HCC risk. The IVW method identified the causal association between higher PAG levels and lower HCC risk: OR, 0.66; 95% CI, 0.462-0.943; P = 0.022.

Table 1 showed the sensitivity analyses including pleiotropy, heterogeneity and horizontal pleiotropy between PAG levels on HCC risk. Scatter plot in Figure 7C visualized the causal effect of PAG levels on HCC risk, respectively. Leave-one-out analysis showed that the correlation of PAG levels with HCC risk was not driven by any single SNP (Figure 7F).

### Analyses of mediating effects

**Figure 8:**
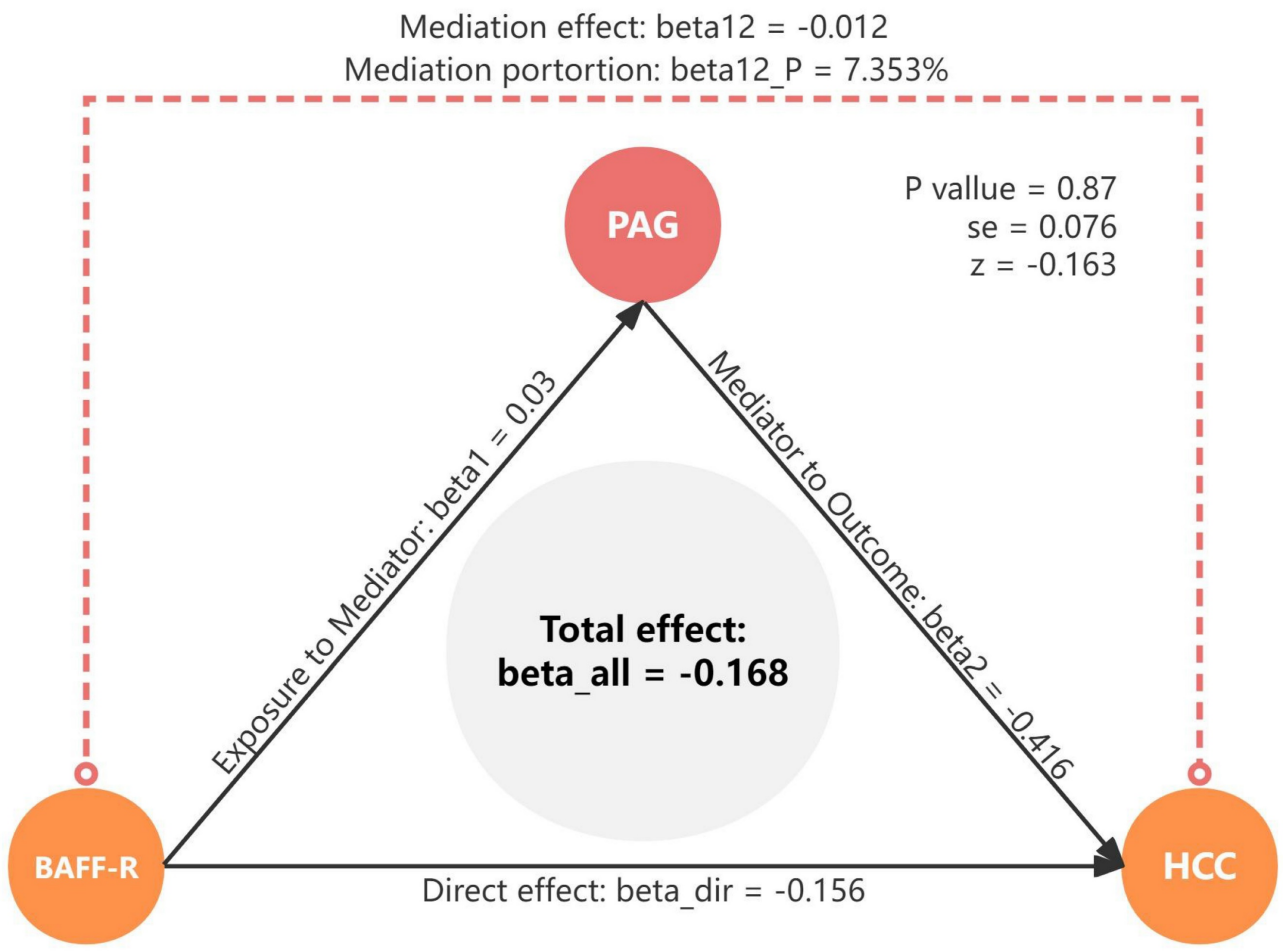
Mediation effect of PAG levels in the association between BAFF-R on IgD+ CD24- B cell and HCC. HCC, hepatocellular carcinoma; PAG, phenylacetylglutamate.

We analyzed the role of PAG levels as a mediator in the pathway from BAFF-R on IgD+ CD24- B cell to HCC (Figure 6). The results indicated that BAFF-R on D+ CD24- was associated with reduced PAG levels, which in turn correlated with a decreased risk of HCC. Figure 8 illustrated that PAG levels contributed to 7.353% of the mediating effect of BAFF-R on IgD+ CD24- B cell in reducing the risk of HCC (P value = 0.87, se = 0.076, and Z = -0.163).

## Discussion

In our study, we presented a characteristic profile of immune cells in HCC. In total, 731 immune cell traits were examined as evidence of HCC risk, with 26 immunophenotypes identified as having potential suggestive associations on the risk of HCC among 6 panels (TBNK, maturation stages of T-cell, Treg, cDC, myeloid cell, and B cell) and 3 traits (MFI, RC, and AC). The increase in immune cell traits, including Plasmacytoid DC %DC, CD20 on CD20- CD38-, BAFF-R on IgD+ CD24- and BAFF-R on IgD- CD24-, may have a palliative phenotype on HCC, as evidenced by significant results obtained from all three MR methods and BWMR method. BWMR analyses provided a more robust validation of the results from MR analyses. A sensitivity test of the MR results was conducted to ensure the reliability and robustness of the research findings. Figure 4 and Figure 5 demonstrated that there was no evidence of horizontal multiplicity in the MR results. Furthermore, the sensitivity test of the Leave-one-out method confirmed the stability of the effect values for all SNPs. The diverse subsets and functions of immune cell traits in the TME of HCC had varying roles.

Plasmacytoid dendritic cells (pDCs) are a distinct subset of dendritic cells known for their capacity to generate significant quantities of type I interferon (IFN-I/α) 23. Typically, pDCs stimulate innate and adaptive immune responses by triggering a cascade of induced reactions in various immune cells within the body, resulting in antigen presentation, differentiation of antibody-producing plasma cells, and ultimately the production of IFN-α. Type I IFN is approved for the clinical treatment of HBV infection. Since the 1990s, IFN-α has been utilized in the treatment of HCC due to its therapeutic efficacy and anti-cancer effects 24-26. However, in the context of cancer, cancer cells exploit the immune-tolerant nature of pDCs to create an immunosuppressive TME that facilitates tumor growth by diminished IFN-α release and increased expression of immune checkpoint regulators 27. Nonetheless, the pDC-targeted vaccine model was utilized in studies to optimally cross-activate CD8 T cells through the transfer of antigens by pDC-derived exosomes, thereby highlighting the key role played by pDC in anti-tumor CD8T cell immunity and in the regulation of the anti-tumor effect of immunotherapy 28. Consistently, our MR results have proven that Plasmacytoid DC %DC is a causally protective factor in HCC. Thus, pDC is expected to be a potential target for HCC therapy.

It has been reported that compared with non-tumor liver tissues, the abundance of all B cell subsets was decreased in the TME, highlighting the anti-tumor role of B cells in human HCC 29. In the “CD20 on CD20- CD38-” trait, CD20, as a CD molecule expressed by B cells, is utilized to assess the level of CD20- CD38- B cells based on mean fluorescence intensities. CD20- CD38- B cells are considered to be a class of naive B cells 17. According to reports, high densities of tumor-infiltrating naive B cells were linked to improved survival in HCC patients 29. A recent study found that high infiltration of CD20+ B cells was associated with poor survival in HCC 30. These findings suggested the potential anti-tumor effects of B cells in the TME of HCC. Our study found that both BAFF-R on IgD+ CD24- B cell and BAFF-R on IgD- CD24- B cell were casually associated with HCC and may reduce the risk of HCC. CD24 expression is dynamic during the lifespan of mature B cells and will lost when B cells begin to differentiate to produce antibodies 31. IgD is an immunoglobulin on the surface of mature B cells. Both the phenotype of BAFF-R on IgD+ CD24- B cell or the phenotype of BAFF-R on IgD- CD24- B cell represented a subset of B cells that express BAFF-R.

BAFF-R, a crucial member of the tumor necrosis factor (TNF) receptor superfamily, promotes the maturation, proliferation, and survival of B cells 32. The expression of BAFF-R collaborated with the quantity of B cells to modulate the steady-state concentrations of BAFF 33. BAFF has a beneficial effect on malignant cells 34. Progressive HCC, large tumor size, and advanced cancer stage may significantly elevate plasma BAFF concentrations and lead to resistance to the biological effects of BAFF 35. It is showed that the expression of BAFF-R was reduced in B cells of patients with HBV-related HCC. As a result, the reduction of BAFF-R led to a decrease in BAFF concentration, thereby exerting a protective effect on HCC 35. Besides, Patients exhibiting positive BAFF-R expression in cases of diffuse large B-cell lymphoma demonstrated a significantly elevated rate of complete remission following chemotherapy, in contrast to their counterparts with negative BAFF-R expression within the same disease cohort 36. Our study strongly supports the above conclusions. It may be crucial to focus on BAFF-R as a novel therapeutic target for future treatment of HCC.

Furthermore, mediation MR analysis also revealed the mediating role of PAG levels in the causal association between BAFF-R on IgD+ CD24- B cell and HCC.

To our knowledge, there have been no reports on the associations between BAFF-R on IgD+ CD24- B cells or PAG and HCC. The scarcity of literature in the field may stem from observational bias and the inadequate delineation of microbial and host co-metabolites in HCC. Concurrently, the employment of varied antibody arrays in flow cytometric experiments, tailored to specific research objectives, could introduce heterogeneity in the phenotypic profiling of immune cell subtypes. Nevertheless, the MR analysis employed in our study achieved the unbiased identification of distinct immune cell subtypes within the disease cohort and enabled the precise localization of BAFF-R on B cell to specific B-cell subtypes. Finally, BAFF-R on IgD+ CD24- B cell was identified as having a potential protective effect against HCC.

PAG (PubChem CID: 11579826), also known as N-Phenylacetylglutamic acid or N-Phenylacetylglutamate, is a byproduct of microbial-host phenylalanine co-metabolism in humans. Its specific physiological effects remained largely unexplored, except for some indications from the study of Pedersen's group. PAG was identified as a specific metabolome marker of ischemic heart disease (IHD) by ultra-performance liquid chromatography with tandem mass spectrometry (UPLC-MS/MS). This revealed that the increased abundance of PAG reflected the enhanced degradation of aromatic amino acids phenylalanine and tyrosine through Gut metabolic module (GMM modules MF0027 and MF0026) 37. Phenylalanine and tyrosine share a convergent metabolic pathway *in vivo*. A prospective study has demonstrated that abnormalities in phenylalanine metabolism was associated with an elevated risk of developing HCC 38. Additionally, impaired catabolism of tyrosine has been identified as being clinically significant in the incipient stages of HCC 39. The tyrosine metabolism-related genes and intermediary product in the phenylalanine metabolism pathway were confirmed to be involved in HCC 40-42. Furthermore, the serum concentration of phenylalanine and tyrosine were both significantly increased in HCC patients 43, 44. The literature has indicated the presence of an interactive dialogue between tyrosine metabolism and the tumor immune microenvironment 45, 46. These findings suggested that the imbalances in phenylalanine and tyrosine metabolism in HCC indicated extensive metabolic reprogramming during the development of the disease. Therefore, as a metabolite of phenylalanine and tyrosine metabolism, PAG may be involved in reprogramming of metabolism and immunity in HCC. This is in accordance with our results. We determined that 7.353% of this effect was mediated through PAG between BAFF-R on IgD+ CD24- B cell and HCC. We uncovered a causal link between BAFF-R on IgD+ CD24- B cell and HCC risk, and highlighted PAG as a mediator. This underscores the complex interplay among immune cells, metabolites, and HCC, paving the way for new avenues of research into the roles of the immune system and metabolites in HCC.

MR can reduce the bias caused by confounding factors and prevent the interference of reverse causality, which is the main strength of this study. It also encourages further exploration of the metabolic-immune interplay in HCC, suggesting intriguing possibilities for future immunotherapy and metabolic therapy approaches.

However, there are still several limitations to this study. First, our analysis was conducted using data from the European population; thus, more ethnic groups should be included in the future. Second, the GWAS dataset for HCC had a small number of cases (N_cases = 453) and 7.353% of the mediation proportion is quite low; thus, the sample size needs to be expanded and other mediators must be quantified in future studies. Third, the data utilized in our investigation are all from public databases, necessitating further retrospective and prospective clinic studies to validate and extend our findings. Fourth, enhancing our understanding of immune cells and metabolites in HCC development by gaining molecular insights from *in vivo* and *in vitro* functional experiments is crucial.

## Conclusion

In this study, MR analysis was firstly conducted to provide genetic evidences of causal correlations between Plasmacytoid DC %DC, CD20 on CD20- CD38-, BAFF-R on IgD+ CD24- and BAFF-R on IgD- CD24- and HCC. Specifically, higher levels of BAFF-R in IgD+ CD24- B cells could lower the HCC risk, with PAG acting as a mediator by the mediation MR analysis. These findings offer novel insights into the mechanisms underlying the development and progression of HCC and suggest potential immune targets for treatment.

## Supplementary Material

Supplementary tables.

## Figures and Tables

**Table 1:** Sensitivity analyses of the study

Exposure	Outcome	Pleiotropy		Heterogeneity		Horizontal pleiotropy
		Egger intercept	Intercept P-value	Q	P-value	Global test P-value
Plasmacytoid DC %DC	HCC	0.052	0.179	27.802	0.114	0.26
BAFF-R on IgD- CD24-	HCC	0.006	0.862	22.272	0.271	0.426
CD20 on CD20- CD38-	HCC	0.045	0.298	10.921	0.617	0.441
BAFF-R on IgD+ CD24-	HCC	0.024	0.485	26.075	0.204	0.248
HCC	BAFF-R on IgD+ CD24-	0.016	0.169	48.574	0.749	0.764
BAFF-R on IgD+ CD24-	PAG	-0.014	0.082	19.776	0.535	0.582
PAG	HCC	-0.026	0.696	20.742	0.475	0.511

Note: PAG, phenylacetylglutamate; IVW, inverse variance weighted; HCC, hepatocellular carcinoma

## Abbreviations

MR: mendelian randomization
HCC: hepatocellular carcinoma
IVW: inverse-variance weighted method
MRPRESSO: MR pleiotropy residual sum and outlier
BWMR: bayesian weighted Mendelian randomization
BAFF-R: B cell-activating factor receptors
PAG: phenylacetylglutamate
TME: tumor microenvironment
SNP: single nucleotide polymorphism
IV: instrumental variable
TSMR: two-sample Mendelian randomization
MFI: median fluorescence intensities
AC: absolute cell
MP: morphological parameters
TBNK: T cell, B cell, natural killer cell
Treg: Regulatory T cell
DC: dendritic cell
CLSA: Canadian Longitudinal Study on Aging
LD: linkage disequilibrium
NAFLD: non-alcoholic fatty liver diseases
OR: odd ratio
HBV: hepatitis B virus
SD: standard deviation
pDC: plasmacytoid dendritic cell
IFN-I/α: I interferon
TNF: tumor necrosis factor
IHD: ischemic heart disease
UPLC-MS/MS: ultra-performance liquid chromatography with tandem mass spectrometry
